# Cryo-Electron Tomography of *Candida glabrata* Plasma Membrane Proteins

**DOI:** 10.3390/jof7020120

**Published:** 2021-02-06

**Authors:** Cristina Jiménez-Ortigosa, Jennifer Jiang, Muyuan Chen, Xuyuan Kuang, Kelley R. Healey, Paul Castellano, Nikpreet Boparai, Steven J. Ludtke, David S. Perlin, Wei Dai

**Affiliations:** 1 Hackensack Meridian Health-Center for Discovery and Innovation, 111 Ideation Way, Nutley, NJ 07110, USA; david.perlin@hmh-cdi.org; 2 Department of Cell Biology and Neuroscience, Rutgers, The State University of New Jersey, 604 Allison Road, Piscataway, NJ 08854, USA; jj549@rutgers.edu (J.J.); kxy700@126.com (X.K.); paulcastellano621@gmail.com (P.C.); nkb60@scarletmail.rutgers.edu (N.B.); 3 Institute for Quantitative Biomedicine, Rutgers, The State University of New Jersey, 174 Frelinghuysen Road, Piscataway, NJ 08854, USA; 4 Department of Biochemistry and Molecular Biology, Baylor College of Medicine, 1 Baylor Plaza, Houston, TX 77030, USA; Muyuan.Chen@bmc.edu (M.C.); sludtke@bcm.edu (S.J.L.); 5 Department of Hyperbaric Oxygen, Central South University, Changsha 410008, China; 6 Department of Biology, William Paterson University, 300 Pompton Road, Wayne, NJ 07470, USA; healeyk3@wpunj.edu

**Keywords:** *Candida glabrata*, fungal membrane proteins, cryo-electron tomography (cryoET)

## Abstract

Fungal plasma membrane proteins have long been recognized as targets for the development of antifungal agents. Despite recent progress in experimental approaches and computational structural predictions, our knowledge of the structural dynamics and spatial distribution of these membrane proteins in the context of their native lipid environment remains limited. By applying cryo-electron tomography (cryoET) and subtomogram analysis, we aim to characterize the structural characteristics and spatial distribution of membrane proteins present in *Candida glabrata* plasma membranes. This study has resulted in the identification of the membrane-embedded structure of the fungal H^+^-ATPase, Pma1. Tomograms of the plasma membrane revealed that Pma1 complexes are heterogeneously distributed as hexamers that cluster into distinct membrane microdomains. This study characterizes fungal membrane proteins in the native cellular landscape and highlights the unique potential of cryoET to advance our understanding of cellular biology and biological systems.

## 1. Introduction

Invasive mycoses have become a significant threat to public health, affecting over a billion people globally and causing over 1.1 million deaths per year. Species from the genera *Aspergillus*, *Candida* and *Cryptococcus* are the most common human pathogenic fungi accounting for a wide variety of invasive and superficial fungal infections. Underlying health conditions such as asthma, acquired immunodeficiency syndrome (AIDS), diabetes, cancer, organ transplantation, and use of corticosteroid therapy are important risk factors for invasive disease. Timely and appropriate antifungal treatment is crucial for the successful outcome of invasive fungal infections [1,2,3,4].

**Figure 1:**
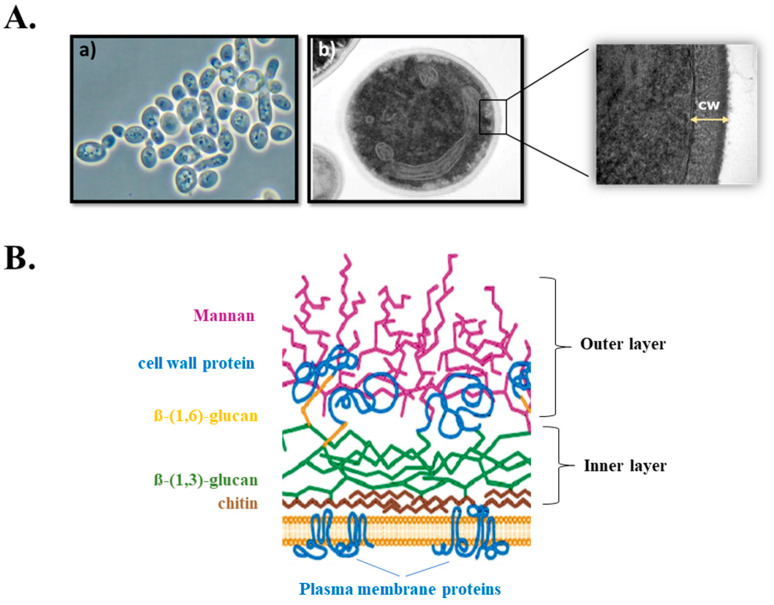
(**A**) *Candida glabrata* cells under (**a**) scanning electron microscope and (**b**) transmission electron microscope (CW = cell wall). (**B**) Schematic representation of the composition and organization of the major components of the fungal cell wall in *Candida* spp. (Figure adapted from [6]).

The fungal cell wall is an essential dynamic structure that undergoes extensive remodeling necessary for growth, survival, fungal morphogenesis and pathogenesis, and protection against osmotic and mechanical stresses. Since the fungal cell wall does not have a mammalian counterpart, it is an excellent target for new antifungal therapies [5]. Fungal cell walls are formed by a complex matrix of polysaccharides and proteins covalently cross-linked to one another, and are organized into layers: a more structured, homogeneous inner layer composed of chitin and β-(1,3)-glucan, and a more heterogeneous outer layer whose composition varies with the growth stage and the fungal species [6] (Figure 1). β-(1,3)-glucan, the most abundant structural component of the fungal cell wall, is synthesized by an enzyme complex whose catalytic subunit is embedded in the plasma membrane and presumably acts as a pore for extrusion of newly synthesized linear β-(1,3)-glucan chains into the cell wall where they assemble and are further modified [7]. Other cell wall components, such as chitins, are also produced by the action of membrane-embedded enzyme complexes [6,8]. The plasma membrane H^+^-ATPase Pma1 is another predominant fungal membrane protein that is responsible for maintaining the electrochemical proton gradients required for nutrient uptake and pH regulation [9]. These protein complexes are critical to the development and physiology of growing fungal cells and have attracted attention as promising therapeutic candidates against invasive mycoses.

*Candida glabrata* has emerged as a common cause of life-threatening fungal infections in many clinical settings in the United States due to its ability to acquire resistance to widely used azole antifungals [10]. As such, the echinocandin antifungals (caspofungin, anidulafungin, and micafungin) are now the preferred front-line therapy against *C. glabrata* and other *Candida* species [11]. They alter the cell wall integrity via inhibition of the catalytic subunit of the enzymatic complex β-(1,3)-glucan synthase (GS), which is encoded by the *FKS1* and *FKS2* genes [12]. Inhibitors targeting chitin synthase and H^+^-ATPases have also been identified [13,14]. These compounds, however, have yet to receive approval for clinical use. The scarcity of antifungal drug classes and the emergence of multidrug resistance to currently available drugs highlight the urgent need for the development of new and effective antifungal agents. Therefore, in situ structural insights are needed to elucidate the mechanisms underlying the functional activity of these fungal membrane proteins and provide the structural basis for the rational design of novel antifungals.

Despite the universal role of membrane proteins in various cellular functions, sample purification of these proteins remains a key bottleneck to unraveling their architecture. Conventional structural techniques either require extraction of the protein of interest from its native environment or membrane protein reconstitution in nanodiscs and liposomes [15,16,17]. Cryo-electron tomography (cryoET) has become a preferred method to characterize the structure of membrane proteins, particularly those that are difficult to purify or crystallize [18,19]. One advantage of cryoET is the ability to directly visualize the cellular landscape and determine the structural dynamics and spatial organization of biomolecules and macromolecular machines from various organisms within their native environment [20,21,22]. Advances in cryoET technology and subtomogram averaging have enabled structural exploration and detailed characterization of cellular processes in yeast cells [23,24,25]. Here, we apply cryoET and subtomogram analysis to determine the structure and spatial distribution of plasma membrane proteins in *C. glabrata* with an initial focus on GS. We observed clusters of ring-like structures heterogeneously distributed on plasma membranes that were enriched following overexpression of GS. However, with structural insights from AlphaFold structure prediction and recently reported high-resolution structures of fungal membrane proteins, the ring-like structures are more appropriately identified as the fungal H^+^-ATPase, Pma1 [26,27,28,29,30,31]. Our structural study into *C. glabrata* offers a new perspective on the dynamics of fungal membrane proteins within the cellular environment and demonstrates the capabilities and versatility of cryoET for cellular structure determination.

## 2. Materials and Methods

### 2.1. Strains

*Candida glabrata* 2001/CBS138 and 200989 (CBS138 his-/trp-/ura-) strains were obtained from the American Type Culture Collection (ATCC, Manassas, VA, USA) and the *FKS1* gene knockout (200989∆*fks1*::*ScURA3*) was a gift from S. Katiyar (Drexel University College of Medicine) [32]. A gap-repair approach, as described in [33], was used to constitutively overexpress *C. glabrata FKS1*. Plasmid pCN-PDC1 (contains nourseothricin resistance marker, a strong promoter (*PDC1*) and *C. glabrata CEN/ARS*) [34] was linearized with EcoRV and treated with alkaline phosphatase (New England Biolabs, Ipswich, MA, USA). The coding region of *FKS1* was amplified with primers that contain overhang regions homologous to each side of the EcoRV restriction site (Table 1). Competent yeast cells were then co-transformed with the purified PCR product and EcoRV-linearized pCN-PDC1. Following the transformation, cells were subjected to a 3-h outgrowth in YPD broth followed by selection on YPD agar medium supplemented with 100 µg/mL nourseothricin (Jena Bioscience, Jena, Germany). All transformants were PCR screened for construct presence and the entire *FKS1* gene insert was subsequently sequenced to confirm wild type sequence (see Table 1 for primers). The ∆*fks1* transformants were also screened for susceptibility to FK506, an *FKS2* inhibitor, (Invivogen, San Diego, CA, USA) to ensure proper *FKS1* expression from plasmid (p*FKS1*). Cells that express *FKS1* exhibit FK506 resistance while the ∆*fks1* parental cells are susceptible [32]. For controls, wild type and ∆*fks1* cells were also transformed with the empty vector.

### 2.2. RNA Extraction and Quantitative RT-PCR

Cells were grown in YPD or YPD supplemented with 100 µg/mL nourseothricin (plasmid-carrying strains) to mid-logarithmic phase. Total RNA was extracted using the RNeasy Mini kit (Qiagen Science, Germantown, MD, USA) according to the manufacturer’s instructions and stored at −80 °C. The concentration and purity of the RNA was determined using a UV spectrophotometer (NanoDrop One; Thermo Fisher Scientific, Waltham, MA, USA) by measuring the absorbance at 230 (OD230), 260 (OD260) and 280 nm (OD280). The integrity of the RNA was further checked by electrophoresis through 1% denaturing and nondenaturing agarose gels. *FKS1* and *FKS2* expression levels were measured by RT-PCR.

All qPCR reactions were performed in a 25-μL reaction mixture consisting of 12.5 μL of 2X One Step RT-PCR buffer (One Step SYBR Ex Taq qRT-PCR kit; TaKaRa Bio Inc., Mountain View, CA, USA), 0.2 μM of each primer, 0.5 μL Takara Ex Taq HS (5 U/μL), 0.5 μL RT Enzyme Mix, and 2 μL of RNA (5 ng/μL) on an Mx3005P real-time instrument (Stratagene, La Jolla, CA, USA). Optimal thermal cycling conditions consisted of 42 °C for 5 min for the reverse transcription, followed by an initial denaturation step at 95 °C for 10 s, 40 cycles of 95 °C for 5 s (denaturation), 60 °C for 20 s (annealing and extension). The experiments were carried out in triplicate for each data point. The relative quantification in gene expression was determined using the 2^−ΔΔCt^ method [35] with expression level of the gene *RDN5.8* for normalization [36]. The primers used are listed in Table 1.

### 2.3. Western Blotting

Fks1 expression levels were determined in total cellular extracts by Western blot analysis as described previously [37] from the following strains CBS138, 200989 ∆*fks1*, 200989 + pCN-PDC1-*FKS1* and 200989 ∆*fks1* + pCN-PDC1-*FKS1*. Blotted proteins were incubated with anti-Fks1 primary antibody (GenScript Biotech, Piscataway, NJ, USA) at a dilution of 1:5000 in 2% TBST at 4 °C for 16 h. Washed membranes were incubated with horseradish peroxidase-conjugated secondary antibody (anti-rabbit; Cell Signaling Technology, Boston, MA, USA) at 1:3000 dilution for 1 h. Bands were visualized with Novex ECL Chemiluminescent substrates (Thermo Fisher Scientific, Waltham, MA, USA) following the manufacturer’s instructions.

### 2.4. Isolation of Enriched Plasma Membrane Fractions

Enriched plasma membrane fractions were isolated from CBS138 (wild type, WT) and 200989 ∆*fks1* + pCN-PDC1-*FKS1* (KH238) strains. Cells were grown in YPD (yeast extract 1%, peptone 2%, dextrose 2%) or YPD supplemented with 100 µg/mL nourseothricin for the plasmid-carrying strain, to mid-logarithmic phase. The cells were collected by centrifugation, washed twice with water and incubated 1 h at 30 °C with 1% ß-mercaptoethanol (MilliporeSigma, Burlington, MA, USA). After incubation, cells were washed twice with water and resuspended in buffer S (1M sorbitol, 10 mM HEPES, pH 6.5). To remove the cell wall and generate protoplasts, lytic enzymes from *Trichoderma harzianum* (MilliporeSigma, Burlington, MA, USA) were added to buffer S, following an incubation overnight at room temperature with gentle shaking. The generation of protoplasts was monitored under the microscope, to assure that at least 90% of the cells lacked the cell wall. The protoplasts were collected by centrifugation and washed twice with PBS1X supplemented with a phosphatase and protease inhibitor cocktail (MilliporeSigma, Burlington, MA, USA) to lyse them and then, loaded into a one-step sucrose gradient. Purified plasma membranes were recovered at the 53.5–43.5% (wt/wt) sucrose interface of a step gradient containing 1 mM EDTA, 1 mM DTT, and 10 mM Tris (pH 7.0) after centrifugation for 3 h at 39,000 rpm in a SW41 rotor (Beckman Coulter, Brea, CA, USA). The membranes were washed for 1 h at 39,000 rpm in a 50.2 Ti rotor (Beckman Coulter, Brea, CA, USA) and resuspended in PBS1X with a phosphatase and protease inhibitors, kept at 4 °C and then plunge frozen before visualization under the cryo-electron microscope.

### 2.5. Preparation of EM Grids

The enriched plasma membrane fractions obtained from wild type and KH238 strains were mixed with 6 nm gold particles as fiducial markers to facilitate tilt series alignment during image processing. An aliquot of 3.5 μL of extracted plasma membrane samples was applied to glow discharged Quantifoil holey grids (R2.0/1.0, Cu, 200 mesh; Quantifoil) prior to vitrification using a Leica EM GP plunger (Leica Microsystems, Buffalo Grove, IL, USA) in a humidity (95%) and temperature (20 °C) controlled chamber. Plunge-frozen grids were stored in a liquid nitrogen dewar until imaging.

### 2.6. Tomography Data Collection

Images and tilt series of the samples were collected on a Talos Arctica cryo-electron microscope (Thermo Fisher Scientific, Waltham, MA, USA) operated at 200 kV, equipped with a post-column BioQuantum energy filter (the slit was set to 20 eV) and a K2 direct electron detector. Automated data collection was performed using SerialEM [38] under the following conditions: 49,000× microscope magnification, spot size 8, 100-μm condenser aperture, and defocus range of −5–−3 μm. The image pixel size was 2.73 Å/pixel. Tilt series ranged from −69° to 69° at 3° step increments. A total of 625 and 272 tilt series of plasma membranes from wild type and KH238 strains were collected, respectively, in counting mode with a cumulative dose of 60–80 e^−^/Å^2^. At each tilt angle, 10 dose fractionation frames were collected to correct for stage drift and beam-induced motion during exposure. A subset of tilt series of wild type yeast plasma membranes was collected from the Thermo Fisher Titan Krios microscope at Purdue University at 42,000× microscope magnification, and defocus at −0.5 μm with Volta phase plate. The image pixel size was 2.80 Å/pixel. This subset was used for generation of an initial model for symmetry analysis and preliminary structural analysis.

### 2.7. Tomography Data Processing

Movie frames for each tilt series were aligned using UCSF MotionCor2 [39]. Alignment of motioned-corrected tilt series and reconstruction of tomograms were performed using the latest EMAN2 tomography workflow [40]. For wild type plasma membranes, tomograms with strong image contrast and detectable ring-like structures were selected for subsequent subtomogram averaging and analysis. From these selected tomograms, a *de novo* initial model was generated with ~50 extracted particles using a box size of 128^3^ pixels. To achieve isotropic resolution for the *de novo* initial model, a subset of subvolumes with side views of the ring-like structures in context of membrane features was included to compensate for the preferred orientation of the particles due to membrane geometry on grids. Five iterations of the EMAN2 reference-free initial model generation routine were performed with no symmetry specified. After alignment to the symmetry axis, the initial model was used for subsequent subtomogram refinement using a larger dataset of 1818 particles. Rotational cross-correlation analysis was performed on the initial average map to assess structural symmetry. 2D plots of coefficients showed signature peaks indicative of C6 symmetry. A second round of refinement was performed with C6 symmetry specified during subtomogram refinement and averaging.

Plasma membranes isolated from Fks1-overexpressing cells featured an apparent increase in the abundance of the larger ring-like structures. A larger set of 5108 particles was extracted for subtomogram averaging. Following a similar subtomogram averaging and analysis procedure as detailed above, with a box size of 168^3^ pixels, a final map with a global resolution of 14 Å was achieved as determined by the Fourier shell correlation (FSC) of density maps from two independent halves of the entire dataset [41].

Visualization, segmentation and domain analysis of 3D maps and subunits were done using Chimera (University of California, San Francisco) [42]. The structure of *Saccharomyces cerevisiae* Pma1 (EMD-31987) was low-pass filtered to 11 Å and fitted into our subtomogram average using the *Fit-in-Map* tool in Chimera [30,42].

### 2.8. Spatial Distribution Analysis

Tomograms containing >250 ring-like structures were used to evaluate the spatial distribution of these particles within the clusters. Coordinates of the center of the ring-like structures were used for subsequent nearest neighbor analysis. Within a cluster, the nearest neighbor of a particle was defined as the particle within the shortest linear distance. The nearest neighbor distance was computed as the distance from the center of a particle to the center of its nearest neighbor. For 2D average analysis, 183 subtomograms containing patches of ring-like structures were extracted using a box size of 256^3^ pixels and then subjected to a 30 Å low pass filter to remove high frequency noises. A 2D average was generated by reference-free 2D refinement using the projections of the 3D subtomograms of these protein complex clusters. Unit cell annotations were done using the measurement functionality available in EMAN2.

## 3. Results

### 3.1. Identification of Two Populations of Ring-like Structures in C. glabrata Plasma Membranes

**Figure 2:**
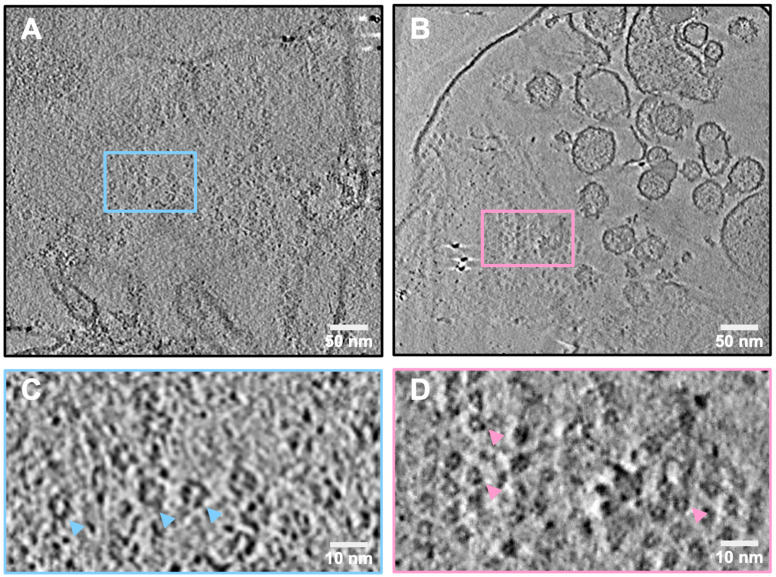
Cryo-electron tomography (cryoET) revealed two ring-like structures in plasma membranes of *Candida glabrata* CBS138 strain. (**A**) Slice view of plasma membranes with clusters of loosely packed, 170 Å diameter large ring-like structures. (**B**) Slice view of a representative tomogram showing the small 125 Å ring-like structures. Electron-dense particles are gold fiducials. (**C**) Zoomed-in slice view of the large ring-like structures (blue arrowheads) boxed in (**A**). (**D**) Zoomed-in slice view of the small ring-like structures (pink arrowheads) boxed in (**B**).

To examine the protein structures present in *C. glabrata* plasma membrane, we first generated protoplasts, which are cell wall-less yeast cells that are viable when incubated in an osmotically stabilizing liquid nutrient medium and can synthesize a new cell wall and revert to a normal phenotype. From the protoplasts of *C. glabrata* wild type strain CBS138, we collected the enriched plasma membrane fraction from a sucrose gradient and imaged these membrane fragments using the Talos Arctica cryo-electron microscope. We examined over 600 tomograms and found that 8–10% contained clusters of a ring-like structure with an approximate diameter of 170 Å. These clusters were heterogeneously distributed on the plasma membranes, and primarily found in patches across large membrane regions (Figure 2A,C; blue arrowheads, and Supplementary Movie S1). In addition, a smaller ring-like structure, with a diameter of 125 Å, was observed in less frequent and more loosely packed clusters (Figure 2B,D; pink arrowheads, and Supplementary Movie S2). The smaller rings were markedly less abundant compared to the larger ring-like structures and were detected in less than 1% of all tomograms.

**Figure 3:**
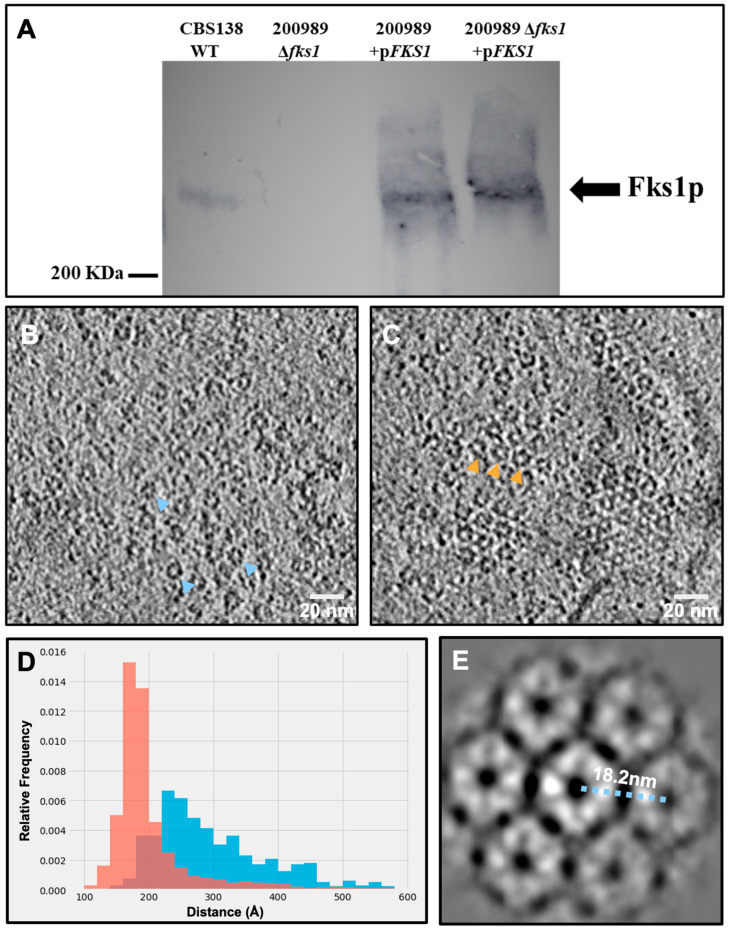
Large ring-like structures in *Candida glabrata* overexpressing Fks1 strain. (**A**) Expression levels of Fks1. *C. glabrata* cells from the different strains were grown on liquid YPD with (plasmid-carrying strains) or without 100 µg/mL nourseothricin until mid-log phase, and proteins were extracted using the TCA method. p*FKS1* = pCN-PDC-*FKS1* and KH238 strain = 200989 Δ*fks1+* p*FKS1*. (**B**) Slice view of a representative tomogram showing a patch of the large ring-like structures from the wild type strain (blue arrowheads). (**C**) Slice view showing clusters of the large ring-like structures in membranes from the strain overexpressing Fks1 (KH238) (orange arrowheads). (**D**) Histogram showing nearest neighbor distance of the large ring-like particles within clusters from the KH238 (orange) and wild type (blue) strains. (**E**) 2D average of the semicrystalline array composed of the large ring-like structures.

To determine whether the large or small ring-like structures corresponded to β-(1,3)-glucan synthase (GS), we constructed strains that constitutively overexpress the *FKS1* gene. We first measured *FKS* gene expression to confirm that the strains carrying the plasmid pCN-PDC1-*FKS1* were indeed overexpressing the *FKS1* gene. RNA was isolated from cells harvested in mid-log growth phase and levels of *FKS1* and *FKS2* mRNA were compared to that of the wild type strains CBS138 and 200989. We observed a 3- and 4-fold increase in the expression level of *FKS1* in the plasmid-containing strains 200989 + p*FKS1* and 200989 Δ*fks1* + p*FKS1*, respectively, when compared to the expression levels of both wild type strains. *FKS2* expression levels also increased by 2-fold in the plasmid-containing strains, similar to the levels observed in the knock-out strain (Table 2). Western blot analysis showed that the strains carrying the plasmid contained a higher abundance of the Fks1 protein compared to the wild type strain (Figure 3A). For further analysis, we used the strain 200989 Δ*fks1* + pCN-PDC-*FKS1* (KH238).

To examine whether the overexpression of Fks1 shifts the abundance and distribution of the large or small ring-like structures, we evaluated tomograms collected from the strain overexpressing Fks1 (KH238) and compared the data to those from the wild type sample. Notably, we observed an apparent increase in the abundance of the large ring structure within plasma membranes from the KH238 strain compared to the wild type (Figure 3B, blue arrowheads; Figure 3C, orange arrowheads, and Movie S3). About 22% of tomograms collected from Fks1-overexpressing plasma membranes contained clusters of the large rings. Overexpression of Fks1 also shifted the packing of the large ring-like structures within the clusters. Nearest neighbor analysis revealed that in plasma membranes of wild type cells, large ring-like structures within clusters formed a 2D distribution that followed a Gaussian distribution with a defined peak at 240 Å (Figure 3D; blue). Overexpression of Fks1 shifted the organization of these protein clusters to a narrower distribution with a peak at 180 Å (Figure 3D; orange), which is slightly larger than the measured diameter of these large ring-like densities (~170 Å). 2D averaging of patches of these clusters confirmed the semicrystalline array packing of the large ring-like structures, with minimal spacing between neighboring complexes (Figure 3E). Taken together, biochemical and structural data suggest that these large ring-like structures could be putative GS complexes.

### 3.2. Structural Determination of the Ring-like Structures by Subtomogram Averaging

**Figure 4:**
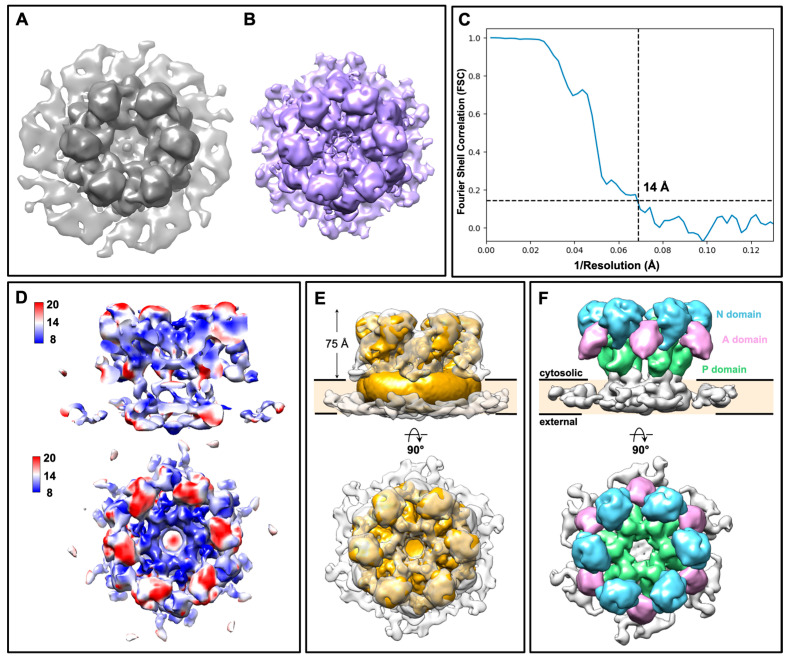
Structural analysis of the ring-like structures as Pma1 hexamers. (**A**) Isosurface, top views of the large ring-like structure from wild type (**A**)—gray and KH238 (**B**)—purple plasma membranes. (**C**) Resolution assessment of the subtomogram average from the Fks1 overexpression strain by Fourier shell correlation (FSC). (**D**) Local resolution evaluation of the subtomogram average from the Fks1-overexpressing strain. (**E**) Fitting of the subtomogram average (gray) with the cryo-electron microscopy (cryoEM) structure of the Pma1 hexamer from *Saccharomyces cerevisiae* (EMD-31987, yellow). (**F**) Segmentation of the subtomogram average showing the three conserved cytosolic domains in the Pma1 monomer: nucleotide-binding (N) domain (blue), phosphorylation (P) domain (green) and actuator (A) domain (pink).

To further investigate whether the ring-like structures are putative GS complexes, we performed subtomogram averaging (Figure S1) with particles extracted from plasma membranes of both wild type and KH238 strains. The overall morphology between the two 3D subtomogram averages from wild type and Fks1-overexpressing cells is similar (Figure 4A,B). Due to the higher apparent abundance of the ring-like structures, a higher resolution subtomogram average was obtained from membranes of the KH238 strain, resolved at ~14 Å as determined by Fourier shell correlation (FSC) (Figure 4C).

The subtomogram average displays dominant C6 symmetry with a central pore measuring 62 Å in diameter (Figure 4B, Movie S4). From local resolution maps, the overall conformation of the complex is relatively stable and rigid with the exception at the top region (Figure 4D; red). Each monomeric unit in the hexameric complex clearly showed an extramembrane density protruding 75 Å towards one side of the membrane, which is significantly higher than that of the AlphaFold predicted structure of GS from *S. cerevisiae* (Figure 4E) [26]. Given the 87% sequence identity between GS from these two fungal species, the ring-like structures are unlikely to correspond to the anticipated GS. The tighter packing of the ring-like structures in the plasma membranes of Fks1-overexpressing cells could be attributed to the overcrowding of protein components in the plasma membrane. Overexpression of the Fks1 protein could influence plasma membrane dynamics and integrity, resulting in the observed increase in the abundance of ring-like structures.

To further clarify the identity of these ring-like structures, we considered the fungal H^+^-ATPase, Pma1, which is one of the most abundant proteins in the yeast plasma membrane. High resolution cryo-electron microscopy (cryoEM) structures of Pma1 from *S. cerevisiae* and *Neurospora crassa* have since been reported, showing that Pma1, unlike most proton pumps in the P-type subfamily, exists as a hexamer [30,31]. Since *C. glabrata* Pma1 shares 89% sequence identity with Pma1 from *S. cerevisiae*, we fitted the *S. cerevisiae* Pma1 cryoEM structure into our subtomogram average, confirming that the ring-like structures are Pma1 hexamers (Figure 4E). The resolution of our subtomogram average was sufficient to assign the extramembrane densities of each monomer to the three conserved cytosolic domains: the nucleotide-binding (N) domain, the phosphorylation (P) domain and the actuator (A) domain (Figure 4F, Movie S4). Interactions between neighboring monomers appear to be mediated by the cytosolic A/N/P-domains. The geometry of plasma membranes on the grids results in preferred orientation, and therefore lower number of side views in our tomograms. Consequently, the transmembrane domain of the Pma1 complex is less well resolved.

## 4. Discussion

CryoET has emerged as a mainstream technique for interrogating the dynamics and function of macromolecular protein complexes in situ. However, this technique comes with inherent challenges. The complexity of the cellular environment complicates structural analysis, particularly the unambiguous identification of proteins within cells. Therefore, specific measures need to be taken to produce a reliable structural analysis and annotation of cellular tomograms. Structural characterization by cryoET often needs to be supported by orthogonal techniques, such as cryo-correlative light and electron microscopy (cryo-CLEM), computational modeling and quantitative mass spectrometry, that provide critical, complementary information. An integrative structural biology paradigm has become important towards achieving a more accurate and comprehensive understanding of complex and dynamic macromolecular protein complexes in their native environment.

Overexpression of *FKS1* to enrich GS for structural studies led to changes in the distribution of other membrane protein populations present in yeast plasma membranes. Although the in situ 3D structure and spatial localization of GS warrant further studies, we were able to characterize the molecular structure and distribution of the fungal proton pump Pma1 embedded in the native lipid environment. In yeast plasma membranes, Pma1 occupies discrete membrane microdomains called membrane compartments of Pma1 (MCPs) [43,44,45]. Appearance of hexagonal clustering of Pma1 in various species of yeast has been previously described [46,47]. Clustering of Pma1 hexamers in tomograms of *C. glabrata* plasma membranes suggests that this higher-order spatial arrangement may be a conserved phenomenon across fungal species and has functional implications.

**Figure 5:**
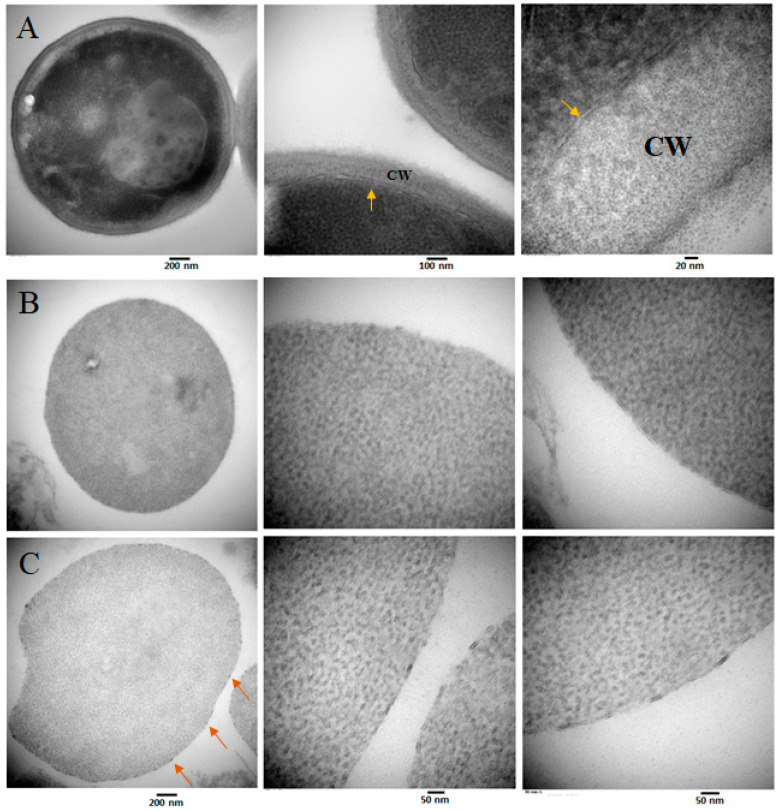
Transmission electron microscopy of (**A**) *Candida glabrata* CBS138 strain growing in log phase. The yellow arrows indicate the plasma membrane. CW = cell wall. (**B**) *C. glabrata* protoplasts after enzymatic removal of the cell wall, and (**C**) regeneration of the *C. glabrata* protoplasts in YPD liquid medium plus sorbitol 1M after 90 min. The orange arrows show electron-dense areas in the plasma membrane, where the synthesis of new components of the cell wall may take place. The size bars for panels B and C are the same.

Other membrane-spanning proteins have been reported to laterally segregate into distinct lipid microdomains within yeast plasma membranes and exhibit characteristic localization patterns [48,49,50,51]. Previous studies in our laboratory have shown that cell wall regeneration may occur in well-defined, electron-dense areas localized throughout the plasma membrane (Figure 5), suggesting that membrane-embedded enzyme complexes involved in cell wall biosynthesis may also organize into distinct membrane compartments. The physiological relevance and molecular mechanisms underlying the distribution of membrane proteins into functionally distinct microdomains remain unclear and require further investigation.

In summary, our findings offer new structural insights into the lateral compartmentalization of fungal membrane proteins in their native membrane environment and highlight the unique capabilities of cryoET to visualize the unperturbed molecular architecture of biological assemblies.

## Figures and Tables

**Table 1:** Oligonucleotides used in this study.

Primer ^†^	Application	Sequence (5′-3′) ^‡^
pCN-PDC1-FKS1F	Gap-repair	CAATTGCCAAAAAACATTAACATCTAGAACTAGTGGATCCCCCGGGCTGCAGGAATTCATGTCTTACAATAATAACGGAC
pCN-PDC1-FKS1R	Gap-repair	AATATTGTTGATGGTGGTAGCTGTGGGTTGTGTTCTCGAGGTCGACGGTATCGATAAGCTTTTATTTGATTGTAGACCAGG
pCN-PDC1F	PCR/sequence	GAGACCAGACTAATACAACTG
pCN-reverse	PCR/sequence	GTTGCCTGCTACGTAAAGTG
CgFKS1c128R	Sequence	GCCATAGCGATGGCATTAGG
CgFKS1c207F	Sequence	CAAGAAATGGTACTTCGCCG
CgFKS1c594F	Sequence	CCTCCTTTGCACCTTTGCAT
CgFKS1c828F	Sequence	TTTACCGTTTTGACTCCTCAC
CgFKS1c999F	Sequence	CCACATGAACTGGAAAACGC
CgFKS1c1214F	Sequence	GAATGCCCTATTACGTGGTG
CgFKS1c1446F	Sequence	GTTGCTTTTCGGTACCGTTG
CgFKS1c1649F	Sequence	GGGTTCTTGAAGGTTTCAACT
CgFKS1expF	qRT-PCR	CAATTGGCAGAACACCGATCCCAA
CgFKS1expR	qRT-PCR	AGTTGGGTTGTCCGTACTCATCGT
CgFKS2expF	qRT-PCR	TACCAACCAGAAGACCAACAGAATGG
CgFKS2expR	qRT-PCR	TCACCACCGCTGATGTTTGGGT
CgRDN5.8F	qRT-PCR	CTTGGTTCTCGCATCGATGA
CgRDN5.8R	qRT-PCR	GGCGCAATGTGCGTTCA

^†^ Numbers in primer names correspond to amino acid location within the coding region (c). ^‡^ Underlined regions of gap-repair cloning primers correspond to *C. glabrata FKS1* sequences, and nonunderlined regions correspond to sequences on pCN-PDC1 surrounding EcoRV restriction site.

**Table 2:** Expression profiling of the *FKS* genes compared to the wild-type strains CBS138 and 200989.

Strain	*FKS1* Fold-Change	*FKS2* Fold-Change
200989 Δ*fks1*	0	2.16 ± 0.22
200989 + pCN-PDC1-FKS1	2.72 ± 0.14	1.85 ± 0.46
200989 Δ*fks1* + pCN-PDC1-*FKS1* (KH238)	4.22 ± 0.72	2.40 ± 0.82

## Data Availability

The data that support the findings of this study are available from the corresponding authors upon reasonable request. Programs used for tomographic data analysis are available from EMAN2.org. Electron density maps of the Pma1 hexamer from wild type and KH238 strains have been deposited in the EMDataBank—accession numbers EMD-23122 and EMD-23123, respectively.

## Supplementary Materials

The following are available online at https://www.mdpi.com/2309-608X/7/2/120/s1. Figure S1. Subtomogram analysis and averaging workflow; Movie S1. Slice views of large ring-like structures from *C. glabrata* CBS138 strain protoplast membrane; Movie S2. Slice views of small ring-like structures from *C. glabrata* CBS138 strain protoplast membrane; Movie S3. Slice views of large ring-like structures from *C. glabrata* KH238 strain protoplast membrane; Movie S4. 3D isosurface representation of subtomogram average of the Pma1 hexamer. The map was displayed with different thresholds and rotation. Subunits of the protein complex were segmented and annotated in different colors.
