# Correction: Jiménez-Ortigosa et al. Cryo-Electron Tomography of *Candida glabrata* Plasma Membrane Proteins. *J. Fungi* 2021, *7*, 120

**DOI:** 10.3390/jof10030201

**Published:** 2024-03-06

**Authors:** Cristina Jiménez-Ortigosa, Jennifer Jiang, Muyuan Chen, Xuyuan Kuang, Kelley R. Healey, Paul Castellano, Nikpreet Boparai, Steven J. Ludtke, David S. Perlin, Wei Dai

**Affiliations:** 1 Hackensack Meridian Health-Center for Discovery and Innovation, 111 Ideation Way, Nutley, NJ 07110, USA; david.perlin@hmh-cdi.org; 2 Department of Cell Biology and Neuroscience, Rutgers, The State University of New Jersey, 604 Allison Road, Piscataway, NJ 08854, USA; jj549@rutgers.edu (J.J.); kxy700@126.com (X.K.); paulcastellano621@gmail.com (P.C.); nkb60@scarletmail.rutgers.edu (N.B.); 3 Institute for Quantitative Biomedicine, Rutgers, The State University of New Jersey, 174 Frelinghuysen Road, Piscataway, NJ 08854, USA; 4 Department of Biochemistry and Molecular Biology, Baylor College of Medicine, 1 Baylor Plaza, Houston, TX 77030, USA; Muyuan.Chen@bmc.edu (M.C.); sludtke@bcm.edu (S.J.L.); 5 Department of Hyperbaric Oxygen, Central South University, Changsha 410008, China; 6 Department of Biology, William Paterson University, 300 Pompton Road, Wayne, NJ 07470, USA; healeyk3@wpunj.edu

## Error in Article Title

The authors wish to update the article title to “Cryo-Electron Tomography of *Candida glabrata* Plasma Membrane Proteins”. This change is necessary to align the title with the corrected annotation and updated findings.

## Error in Abstract

We adjusted the focus of the abstract to cover the correct annotation of the ring-like structures as Pma1. These changes ensure that the abstract accurately reflects the updated annotation and aligns with corresponding changes in the main text. The new abstract is shown below.

Fungal plasma membrane proteins have long been recognized as targets for the development of antifungal agents. Despite recent progress in experimental approaches and computational structural predictions, our knowledge of the structural dynamics and spatial distribution of these membrane proteins in the context of their native lipid environment remains limited. By applying cryo-electron tomography (cryoET) and subtomogram analysis, we aim to characterize the structural characteristics and spatial distribution of membrane proteins present in *Candida glabrata* plasma membranes. This study has resulted in the identification of the membrane-embedded structure of the fungal H^+^-ATPase, Pma1. Tomograms of the plasma membrane revealed that Pma1 complexes are heterogeneously distributed as hexamers that cluster into distinct membrane microdomains. This study characterizes fungal membrane proteins in the native cellular landscape and highlights the unique potential of cryoET to advance our understanding of cellular biology and biological systems.

## Error in Keywords

We replaced “glucan synthase (GS)” with “fungal membrane proteins” since GS is no longer the only highlighted term in the corrected publication. 

## Error in Figure 4E

In the original publication [1], there was an error in Figure 4E. Given recent publications on fungal membrane proteins [2,3,4,5,6] and insights from AlphaFold structure prediction [7], the hexameric structure, initially proposed as β-(1,3)-glucan synthase (GS), appears to map more closely to the fungal proton pump, Pma1. To support the identity of this structure, we performed fitting of the cryo-electron microscopy (cryoEM) structure of the Pma1 hexamer from *Saccharomyces cerevisiae* with our subtomogram average. We wish to update Figure 4 in the original publication with the structure fitting and corrected domain annotation. The authors state that the resolved structure and distribution analyses remain valid. We regret any confusion or inconvenience caused. The main text and Supplementary Movie S4 in the original publication have also been updated accordingly.

## Error in Introduction

We made three modifications as listed below:

1. In light of the revised annotation, we added a few sentences at the end of the second paragraph (see below) to introduce Pma1 and chitin synthase to maintain the logical flow and coherence of the corrected publication. These changes are necessary to set the stage for subsequent discussion on the revised structural analysis.

Other cell wall components, such as chitins, are also produced by the action of membrane-embedded enzyme complexes [6,8]. The plasma membrane H^+^-ATPase Pma1 is another predominant fungal membrane protein that is responsible for maintaining the electrochemical proton gradients required for nutrient uptake and pH regulation [9]. These protein complexes are critical to the development and physiology of growing fungal cells and have attracted attention as promising therapeutic candidates against invasive mycoses.

2. At the end of the third paragraph, we replaced discussion of GS with elaboration of Pma1 and other fungal membrane proteins. These changes echo the previous paragraph and strengthen the overall coherence of the corrected publication.

Inhibitors targeting chitin synthase and H^+^-ATPases have also been identified [13,14]. These compounds, however, have yet to receive approval for clinical use. The scarcity of antifungal drug classes and the emergence of multidrug resistance against currently available drugs highlight the urgent need for the development of new and effective antifungal agents. Therefore, in situ structural insights are needed to elucidate the mechanisms underlying the functional activity of these fungal membrane proteins and provide the structural basis for the rational design of novel antifungals.

3. At the end of the fourth paragraph, we updated the annotation of the ring-like structure from GS to Pma1 and provided references to recent publications to support the correction.

We observed clusters of ring-like structures heterogeneously distributed on plasma membranes that were enriched following the overexpression of GS. However, with structural insights from the AlphaFold structure prediction and recently reported high-resolution structures of fungal membrane proteins, the ring-like structures are more appropriately identified as the fungal H^+^-ATPase, Pma1 [26–31]. Our structural study of *C. glabrata* offers a new perspective on the dynamics of fungal membrane proteins within the cellular environment and demonstrates the capabilities and versatility of cryoET for use in cellular structure determination.

## Error in Material and Methods

1. In the last paragraph of Section 2.7, we included structural fitting in our analysis to substantiate the updated annotation of the ring-like structures.

The structure of *Saccharomyces cerevisiae* Pma1 (EMD-31987) was low-pass-filtered to 11 Å and fitted into our subtomogram average using the *Fit-in-Map* tool in Chimera [30,42]. 

## Error in Results

We updated some sentences in Section 3.2, which now reads as follows:

### 3.2. Structural Determination of the Ring-like Structures via Subtomogram Averaging

To further investigate whether the ring-like structures are putative GS complexes, we performed subtomogram averaging (Figure S1) using particles extracted from the plasma membranes of both wild type and KH238 strains. The overall morphology between the two 3D subtomogram averages from wild type and Fks1-overexpressing cells is similar (Figure 4A,B). Due to the higher apparent abundance of the ring-like structures, a higher resolution subtomogram average was obtained from membranes of the KH238 strain, resolved at ~14 Å, as determined via Fourier shell correlation (FSC) (Figure 4C).

The subtomogram average displays dominant C6 symmetry with a central pore measuring 62 Å in diameter (Figure 4B, Movie S4). From local resolution maps, the overall conformation of the complex is relatively stable and rigid, with the exception of the top region (Figure 4D; red). Each monomeric unit in the hexameric complex clearly showed an extramembrane density protruding 75 Å toward one side of the membrane, which is significantly higher than that of the AlphaFold-predicted structure of GS from *S. cerevisiae* (Figure 4E) [26]. Given the 87% sequence identity between GS from these two fungal species, the ring-like structures are unlikely to correspond to the anticipated GS. The tighter packing of the ring-like structures in the plasma membranes of Fks1-overexpressing cells could be attributed to the overcrowding of protein components in the plasma membrane. The overexpression of the Fks1 protein could influence plasma membrane dynamics and integrity, resulting in the observed increase in the abundance of ring-like structures.

To further clarify the identity of these ring-like structures, we considered the fungal H^+^-ATPase, Pma1, which is one of the most abundant proteins in the yeast plasma membrane. High-resolution cryo-electron microscopy (cryoEM) structures of Pma1 from *S. cerevisiae* and *Neurospora crassa* have since been reported, showing that Pma1, unlike most proton pumps in the P-type subfamily, exists as a hexamer [30,31]. Since *C. glabrata* Pma1 shares 89% sequence identity with Pma1 from *S. cerevisiae*, we fitted the *S. cerevisiae* Pma1 cryoEM structure into our subtomogram average, confirming that the ring-like structures are Pma1 hexamers (Figure 4E). The resolution of our subtomogram average was sufficient to assign the extramembrane densities of each monomer to the three conserved cytosolic domains: the nucleotide-binding (N) domain, the phosphorylation (P) domain and the actuator (A) domain (Figure 4F, Movie S4). Interactions between neighboring monomers appear to be mediated by the cytosolic A/N/P-domains. The geometry of plasma membranes on the grids results in preferred orientation and, therefore, a lower number of side views in our tomograms. Consequently, the transmembrane domain of the Pma1 complex is less well resolved.

## Error in Discussion

We revised the discussion section to (1) reflect the corrected annotation and its implications, (2) highlight the advantages and pitfalls of employing cryoET for in situ structure determination and (3) offer suggestions on how to effectively address these challenges. These changes ensure that our discussion echoes the updated findings and provides a practical perspective regarding the methodology used in our study. The new discussion is defined below.

CryoET has emerged as a mainstream technique for interrogating the dynamics and function of macromolecular protein complexes in situ. However, this technique comes with inherent challenges. The complexity of the cellular environment complicates structural analysis, particularly the unambiguous identification of proteins within cells. Therefore, specific measures need to be used to produce a reliable structural analysis and annotation of cellular tomograms. Structural characterization by cryoET often needs to be supported by orthogonal techniques, such as cryo-correlative light and electron microscopy (cryo-CLEM), computational modeling and quantitative mass spectrometry, which provide critical, complementary information. An integrative structural biology paradigm has become important for achieving a more accurate and comprehensive understanding of complex and dynamic macromolecular protein complexes in their native environment.

The overexpression of *FKS1* to enrich GS for structural studies led to changes in the distribution of other membrane protein populations present in yeast plasma membranes. Although the in situ 3D structure and spatial localization of GS warrant further studies, we were able to characterize the molecular structure and distribution of the fungal proton pump Pma1 embedded in the native lipid environment. In yeast plasma membranes, Pma1 occupies discrete membrane microdomains called membrane compartments of Pma1 (MCPs) [43,44,45]. The appearance of hexagonal clustering of Pma1 in various species of yeast has previously been described [46,47]. The clustering of Pma1 hexamers in tomograms of *C. glabrata* plasma membranes suggests that this higher-order spatial arrangement may be a conserved phenomenon across fungal species and has functional implications.

Other membrane-spanning proteins have been reported to laterally segregate into distinct lipid microdomains within yeast plasma membranes and exhibit characteristic localization patterns [48,49,50,51]. Previous studies in our laboratory have shown that cell wall regeneration may occur in well-defined, electron-dense areas localized throughout the plasma membrane (Figure 5), suggesting that membrane-embedded enzyme complexes involved in cell wall biosynthesis may also organize into distinct membrane compartments. The physiological relevance and molecular mechanisms underlying the distribution of membrane proteins into functionally distinct microdomains remain unclear and require further investigation.

In summary, our findings offer new structural insights into the lateral compartmentalization of fungal membrane proteins in their native membrane environment and highlight the unique capabilities of cryoET to visualize the unperturbed molecular architecture of biological assemblies.

## Text Correction

We made additional minor edits to improve clarity. These changes do not affect the scientific content. Modifications in specific sections of the publication are shown below. 

1. We replaced “putative GS” with “ring-like” throughout the publication.

**Text Correction in Introduction**

2. In the sentence “Cryo-electron tomography (cryoET) has become a preferred method to characterize the structure of membrane proteins, particularly those that are difficult to purify or crystallize [18,19].”, we changed “the preferred method” to “a preferred method”.

3. We updated the last sentence to read “Here, we apply cryoET and subtomogram analysis to determine the structure and spatial distribution of plasma membrane proteins in *C. glabrata* with an initial focus on GS.”

**Text Correction in Methods and Methods**

4. In the sentence “Plunge-frozen grids were stored in a liquid nitrogen dewar until imaging.”, we deleted the word “flask”.

5. In the sentence “Plasma membranes isolated from Fks1-overexpressing cells featured an apparent increase in the abundance of the larger ring-like structures.”, we replaced “greater” with “an apparent increase in the”.

6. We updated the title of Section 2.8 to “Spatial Distribution Analysis” to align with the corrected annotation.

**Text Correction in Results**

7. In Figures 2 and 3, we replaced “arrows” with “arrowheads”.

8. In the sentence “To determine whether the large or small ring-like structures corresponded to β-(1,3)-glucan synthase (GS), we constructed strains that constitutively express the *FKS1* gene.”, we deleted “the uncharacterized”, since the 3D structure of GS has already been published.

9. We updated the sentence “Notably, the relative abundance of the large ring structure increased significantly in the KH238 strain compared to the wild type.” to “Notably, we observed an apparent increase in the abundance of the large ring structure within plasma membranes from the KH238 strain compared to the wild type”. 

10. In the sentence “Taken together, biochemical and structural data suggest that these large ring-like structures could be putative GS complexes.”, we replaced the word “are” with “could be”.

11. In the legend of Figure 3, we revised the abbreviation “YDP” to “YPD”. We also modified the sentence to read “Slice view showing clusters of the large ring-like structures in membranes from the strain overexpressing Fks1 (KH238) (orange arrowheads).”

**Text Correction in Supplementary Materials**

12. Changed “putative GS complex” to “Pma1 hexamer”.

## References Correction

**Newly added** references show below:

8. Garcia-Rubio, R.; de Oliveira, H.C.; Rivera, J.; Trevijano-Contador, N. The Fungal Cell Wall: *Candida*, *Cryptococcus*, and *Aspergillus* Species. *Front. Microbiol.* **2020**, *10*, 2993.

9. Serrano, R. Structure and function of proton translocating ATPase in plasma membranes of plants and fungi. *Biochim. Biophys. Acta* **1988**, *947*, 1–28.

13. Merzendorfer, H. Chitin synthesis inhibitors: old molecules and new developments. *Insect Sci.* **2013**, *20*, 121–138.

14. Bublitz, M.; Kjellerup, L.; Cohrt, K.O.; Gordon, S.; Mortensen, A.L.; Clausen, J.D.; Pallin, T.D.; Hansen, J.B.; Fuglsang, A.T.; Dalby-Brown, W.; et al. Tetrahydrocarbazoles are a novel class of potent P-type ATPase inhibitors with antifungal activity. *PLoS ONE* **2018**, *13*, e0188620.

26. Jumper, J.; Evans, R.; Pritzel, A.; Green, T.; Figurnov, M.; Ronneberger, O.; Tunyasuvunakool, K.; Bates, R.; Žídek, A.; Potapenko, A.; et al. Highly accurate protein structure prediction with AlphaFold. *Nature* **2021**, *596*, 583–589.

27. Hu, X.; Yang, P.; Chai, C.; Liu, J.; Sun, H.; Wu, Y.; Zhang, M.; Zhang, M.; Liu, X.; Yu, H. Structural and mechanistic insights into fungal β-1,3-glucan synthase FKS1. *Nature* **2023**, *616*, 190–198.

28. Zhao, C.-R.; You, Z.-L.; Chen, D.-D.; Hang, J.; Wang, Z.-B.; Ji, M.; Wang, L.-X.; Zhao, P.; Qiao, J.; Yun, C.-H.; et al. Structure of a fungal 1,3-β-glucan synthase. *Sci. Adv.* **2023**, *9*, eadh7820.

29. Ren, Z.; Chhetri, A.; Guan, Z.; Suo, Y.; Yokoyama, K.; Lee, S.-Y. Structural basis for inhibition and regulation of a chitin synthase from *Candida albicans*. *Nat. Struct. Mol. Biol.* **2022**, *29*, 653–664.

30. Zhao, P.; Zhao, C.; Chen, D.; Yun, C.; Li, H.; Bai, L. Structure and activation mechanism of the hexameric plasma membrane H^+^-ATPase. *Nat. Commun.* **2021**, *12*, 6439.

31. Heit, S.; Geurts, M.M.G.; Murphy, B.J.; Corey, R.A.; Mills, D.J.; Kühlbrandt, W.; Bublitz, M. Structure of the hexameric fungal plasma membrane proton pump in its autoinhibited state. *Sci. Adv.* **2021**, *7*, eabj5255.

43. Malínská, K.; Malínský, J.; Opekarová, M.; Tanner, W. Visualization of Protein Compartmentation within the Plasma Membrane of Living Yeast Cells. *Mol. Biol. Cell.* **2003**, *14*, 4427–4436.

44. Malinska, K.; Malinsky, J.; Opekarova, M.; Tanner, W. Distribution of Can1p into stable domains reflects lateral protein segregation within the plasma membrane of living *S. cerevisiae* cells. *J. Cell Sci.* **2004**, *117*, 6031–6041.

45. Merzendorfer, H.; Heinisch, J. Microcompartments within the yeast plasma membrane. *Biol. Chem.* **2013**, *394*, 189–202.

46. Hippe, S.; Lüth, H. A simple physical model for fungicide induced hexagonal clustering of intramembrane particles in the plasmalemma of *Ustilago avenae*. *J. Theor. Biol.* **1986**, *121*, 351–366.

47. Kübler, O.; Gross, H.; Moor, H. Complementary structures of membrane fracture faces obtained by ultrahigh vacuum freeze-fracturing at -196 degrees C and digital image processing. *Ultramicroscopy* **1978**, *3*, 161–168.

48. Grossman, G.; Opekarová, M.; Malinsky, J.; Weig-Meckl, I.; Tanner, W. Membrane potential governs lateral segregation of plasma membrane proteins and lipids in yeast. *EMBO J.* **2007**, *26*, 1–8.

49. Grossman, G.; Malinsky, J.; Stahlschmidt, W.; Loibl, M.; Weig-Meckl, I.; Frommer, W.B.; Opekarová, M.; Tanner, W. Plasma membrane microdomains regulate turnover of transport proteins in yeast. *J. Cell Biol.* **2008**, *183*, 1075–1088.

50. Bianchi, F.; Syga, Ł.; Moiset, G.; Spakman, D.; Schavemaker, P.E.; Punter, C.M.; Seinen, A.-B.; van Oijen, A.M.; Robinson, A.; Poolman, B. Steric exclusion and protein conformation determine the localization of plasma membrane transporters. *Nat. Commun.* **2018**, *9*, 501.

51. Spira, F.; Mueller, N.S.; Beck, G.; von Olshausen, P.; Beig, J.; Wedlich-Söldner, R. Patchwork organization of the yeast plasma membrane into numerous coexisting domains. *Nat. Cell Biol.* **2012**, *14*, 640–648.

These references **removed**, the list show below:

11. Pfaller, M.A.; Castanheira, M.; Lockhart, S.R.; Ahlquist, A.M.; Messer, S.A.; Jones, R.N. Frequency of decreased susceptibility and resistance to echinocandins among fluconazole-resistant bloodstream isolates of *Candida glabrata*. *J. Clin. Microbiol.* **2012**, *50*, 1199–203.

12. Alexander, B.D.; Johnson, M.D.; Pfeiffer, C.D.; Jiménez-Ortigosa, C.; Catania, J.; Booker, R.; Castanheira, M.; Messer, S.A.; Perlin, D.S.; Pfaller, M.A. Increasing echinocandin resistance in *Candida glabrata*: Clinical failure correlates with presence of *FKS* mutations and elevated minimum inhibitory concentrations. *Clin. Infect. Dis.* **2013**, *56*, 1724–1732.

13. Perlin, D.S. Echinocandin Resistance in *Candida*. *Clin. Infect. Dis.* **2015**, *61*, S612–617.

14. Johnson, M.E.; Edlind, T.D. Topological and mutational analysis of *Saccharomyces cerevisiae* Fks1. *Eukaryot. Cell* **2012**, *11*, 952–960.

37. Rico, H.; Carrillo, C.; Aguado, C.; Mormeneo, S.; Sentandreu, R. Initial steps of wall protoplast regeneration in *Candida albicans*. *Res. Microbiol.* **1997**, *148*, 593–603.

38. Okada, H.; Abe, M.; Asakawa-Minemura, M.; Hirata, A.; Qadota, H.; Morishita, K.; Ohnuki, S.; Satoru Nogami, S.; Ohya, Y. Multiple Functional Domains of the Yeast l,3-β-Glucan Synthase Subunit Fks1p Revealed by Quantitative Phenotypic Analysis of Temperature-Sensitive Mutants. *Genetics* **2010**, *184*, 1013–1024.

39. Okada, H.; Ohnuki, S.; Roncero, C.; Konopka, J.B.; Ohya, Y. Distinct roles of cell wall biogenesis in yeast morphogenesis as revealed by multivariate analysis of high-dimensional morphometric data. *Mol. Biol. Cell.* **2014**, *25*, 222–233.

40. Purushotham, P.; Ruoya, H.; Zimmer, J. Architecture of a catalytically active homotrimeric plant cellulose synthase complex. *Science* **2020**, *369*, 1089–1094.

The order of citations will be adjusted accordingly according to the addition and deletion.

The authors state that the scientific conclusions are unaffected. This correction was approved by the Academic Editor. The original publication has also been updated.

## Figures and Tables

**Figure 4:**
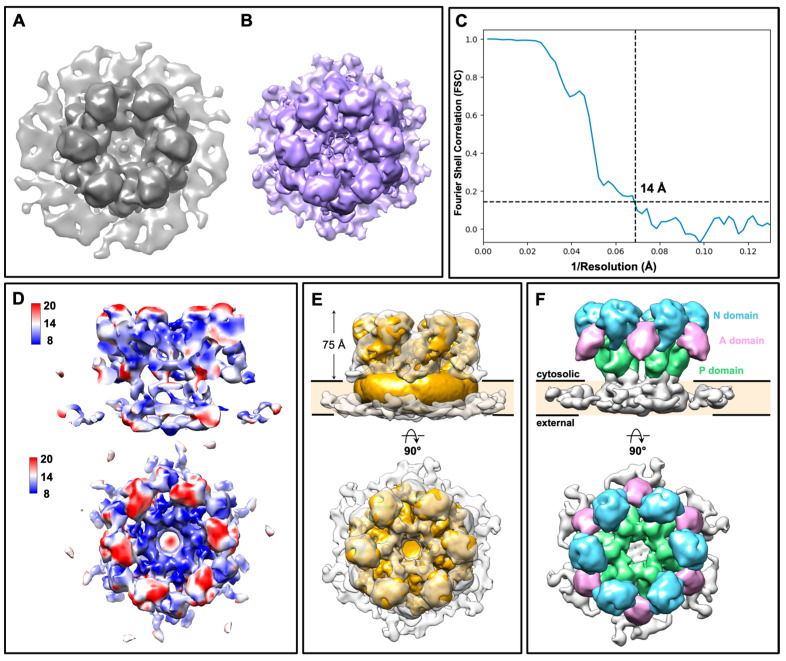
Structural analysis of the ring-like structures as Pma1 hexamers. (**A**) Isosurface, top views of the large ring-like structure from the wild type. (**A**)—gray and KH238 (**B**)—purple plasma membranes. (**C**) Resolution assessment of the subtomogram average from the Fks1 overexpression strain via Fourier shell correlation (FSC). (**D**) Local resolution evaluation of the subtomogram average from the Fks1-overexpressing strain. (**E**) Fitting of the subtomogram average (gray) with the cryo-electron microscopy (cryoEM) structure of the Pma1 hexamer from *Saccharomyces cerevisiae* (EMD-31987, yellow). (**F**) Segmentation of the subtomogram average showing the three conserved cytosolic domains in the Pma1 monomer: nucleotide-binding (N) domain (blue), phosphorylation (P) domain (green), and actuator (A) domain (pink).
