# Supplementary material for: Cryo-Electron Tomography of Candida glabrata Plasma Membrane Proteins
Source: J Fungi (Basel). 2021 Feb 6;7(2):120. doi: 10.3390/jof7020120 (PMC7914498; doi:10.3390/jof7020120)
Supplement: Supplementary file 1 [file jof-07-00120-s001.zip › Figure S1.pdf]

Supplementary materials

Supplementary Figure 1. Subtomogram analysis and averaging workflow.

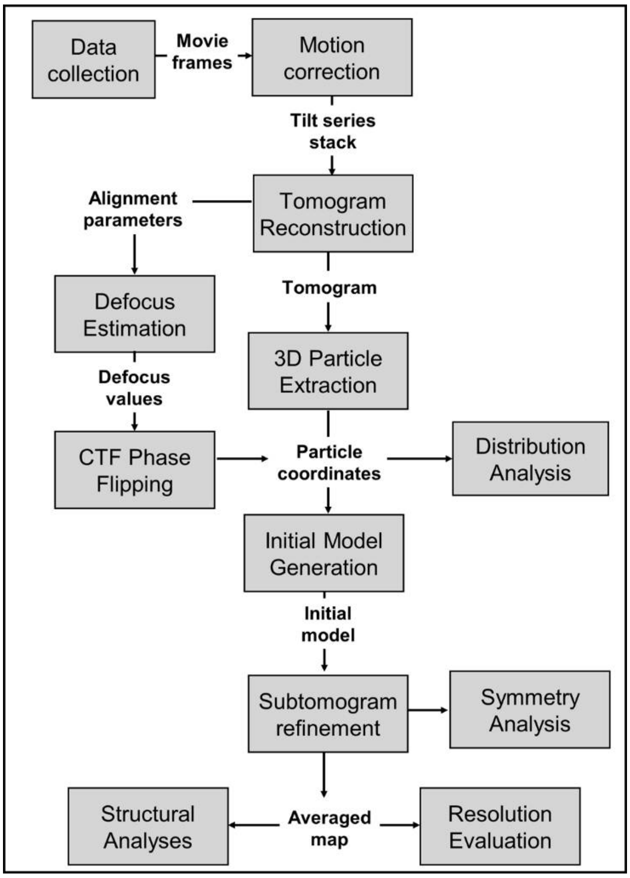

Supplementary Movies

Movie S1. Slice views of large ring-like structures from C. glabrata CBS138 strain protoplast membrane.

Movie S2. Slice views of small ring-like structures from C. glabrata CBS138 strain protoplast membrane.

Movie S3. Slice views large ring-like structures from C. glabrata KH238 strain protoplast membrane.

Movie S4. 3D isosurface representation of subtomogram average of the putative GS complex. The map was displayed with different thresholds and rotation. Subunits of the protein complex were segmented and annotated in different colors.
